# Migraine management in pregnancy

**Published:** 2012-11

**Authors:** Nasrin Jalilian, Taravat Fakheri, Sara Daeichin, Faranak Vakilifar

**Affiliations:** ^*a*^Maternity Research Center, Kermanshah University of Medical Sciences, Kermanshah, Iran.; ^*b*^Bureau chief of English language journal of JIVR, Deputy of Research and Technology, Kermanshah University of Medical Sciences, Kermanshah, Iran.

## Abstract

**Background:**

Migraine and tension-type headache are primary headache disorders that occur during pregnancy. Most women with migraine improve during pregnancy. Some women have their first attack during pregnancy. Migraine can recur postpartum; it can also begin at that time. Women who have had menstrual migraine and migraine onset at menarche tend to experience no migraine during pregnancy. Not all migraines improve during pregnancy, however. Some women experience migraine for the first time during pregnancy.

**Etiology:**

Headaches caused by cerebral arteriovenous malformations often present as migraine with aura. Cerebral venous thrombosis (common during pregnancy and the puerperium) may manifest with migraine-like visual disturbance and headache.

**Treatment:**

Nondrug therapies (relaxation, sleep, massage, ice packs and biofeedback) should be tried first to treat migraine in women who are pregnant. For treatment of acute migraine attacks 1000 mg of paracetamol (acetaminophen) preferably as a suppository is considered the first choice drug treatment.

**Conclusions:**

Migraine has also been recently postulated as one of the major risk factors for stroke during pregnancy and the puerperium. There is thus an urgent need for prospective studies of large numbers of pregnant women to determine the real existence and extent of the risks posed by migraine during pregnancy.

**Keywords:**

Migraine, Pregnancy, Headache


					Volume 4, Suppl. 1 Nov 2012
Publisher: Kermanshah University of Medical Sciences
URL: http://www.jivresearch.org
Copyright:
Congress of Iranian Neurosurgeons, 14-16 November 2012, Kermanshah, Iran
Paper No. 78
Migraine management in pregnancy
Nasrin Jalilian a
, Taravat Fakheri a,*
, Sara Daeichin a
, Faranak Vakilifar b
a
Maternity Research Center, Kermanshah University of Medical Sciences, Kermanshah, Iran.
b
Bureau chief of English language Journal of JIVR, Deputy of Research and Technology, Kermanshah University of Medical
Sciences, Kermanshah, Iran.
Abstract:
Background: Migraine and tension-type headache are primary headache disorders that occur during pregnancy.
Most women with migraine improve during pregnancy. Some women have their first attack during pregnancy.
Migraine can recur postpartum; it can also begin at that time. Women who have had menstrual migraine and
migraine onset at menarche tend to experience no migraine during pregnancy. Not all migraines improve during
pregnancy, however. Some women experience migraine for the first time during pregnancy.
Etiology: Headaches caused by cerebral arteriovenous malformations often present as migraine with aura. Cerebral
venous thrombosis (common during pregnancy and the puerperium) may manifest with migraine-like visual
disturbance and headache.
Treatment: Nondrug therapies (relaxation, sleep, massage, ice packs and biofeedback) should be tried first to treat
migraine in women who are pregnant. For treatment of acute migraine attacks 1000 mg of paracetamol
(acetaminophen) preferably as a suppository is considered the first choice drug treatment.
Conclusions: Migraine has also been recently postulated as one of the major risk factors for stroke during
pregnancy and the puerperium. There is thus an urgent need for prospective studies of large numbers of pregnant
women to determine the real existence and extent of the risks posed by migraine during pregnancy.
Keywords:
Migraine, Pregnancy, Headache
* Corresponding Author at:
Taravat Fakheri: Associated professor of Obs & Gyn Department, Maternity Research Center of Kermanshah University of Medical Sciences,
Kermanshah, Iran, Email: tfakheri@kums.ac.ir, (Fakheri T.).

				

